# Use of beneficial microorganisms in bean cultivation: strategies employed to increase productivity

**DOI:** 10.1007/s42770-026-02074-9

**Published:** 2026-08-31

**Authors:** Matheus Messias, Jerri Édson Zilli, Luis Henrique de Barros Soares, Enderson Petrônio de Brito Ferreira, Veronica Massena Reis

**Affiliations:** 1 Postgraduate Program in Agronomy, Universidade Federal Rural do Rio de Janeiro, Seropédica, Rio de Janeiro Brazil; 2 Embrapa Agrobiologia, Seropédica, Rio de Janeiro Brazil; 3 Embrapa Arroz e Feijão, Santo Antônio de Goiás, Goiás Brazil; 4 Embrapa Clima Temperado, Pelotas, Rio Grande do Sul Brazil

**Keywords:** *Phaseolus vulgaris*, *Rhizobium*, PGPRs, Co-inoculation, Inoculant

## Abstract

The common bean is of great importance for human nutrition worldwide as its grain is a cheap source of protein, but it has a high-cost production system due to the use of synthetic inputs such as nitrogen fertilizers and pest and disease control products. An alternative to promote the sustainability of the production system and increase crop productivity is the use of co-inoculation of plant growth-promoting rhizobacteria (PGPR) to reduce and/or replace the use of nitrogen fertilizers and pest and disease control products. Since 2010, Brazil has stood out on the international stage with research focused on the application of rhizobia combined with PGPR in bean crops. Currently, the use of microorganisms that effectively promote plant growth through different mechanisms of action is already a consolidated reality in Brazil, with experimental results proven by years of research. Despite these advances, there are still challenges to overcome to maximize the potential of co-inoculation in bean crops. These include the need to develop more efficient formulations containing elite microbial strains and optimized microbial consortia, as well as the search for inoculation methods that optimize root colonization by the bacteria and maximize their beneficial effects on the plant. In this review, we discuss the history of common-bean production and the types and doses of inoculants marketed, as well as the challenges for the efficiency of co-inoculation and for the development of inoculants of rhizobial consortia with PGPRs for the common-bean cultivation.

## Introduction

Medium to high-tech production systems of common beans require the efficient use of biological inputs to ensure high yields and reduce environmental impact [1]. In the production system of common bean (*Phaseolus vulgaris* L.), the most necessary nutrient, nitrogen (N), can be provided by the use of inoculants containing bacteria of the genus *Rhizobium*, capable of performing the process of biological nitrogen fixation (BNF). BNF contributes to plant growth and increased grain production, and can partially and/or totally replace the use of nitrogen fertilizers, thus helping to reduce environmental impact [2].

The search for innovation has led to the continuous improvement of inoculants for common beans, increasing their effectiveness and adaptability to soil and climatic conditions in different environments in Brazil, resulting in the approval of the strains of *R. tropici*, SEMIA 4077 (= CIAT 899) and SEMIA 4088 (= H 12), and the strain of *R. freirei*, SEMIA 4080 (= PRF 81), for the production of inoculants for the common bean crop [3].

The single inoculation with *R. tropici* is the most traditional in Brazil, resulting in average grain yields of 2,500 to 3,000 kg ha^− 1^ [2]. However, innovative technologies such as the production of inoculants composed of nitrogen-fixing bacteria, plant growth promoters and phytopathogen controllers are gaining ground [3]. This technology is known as co-inoculation, which involves the synergism of combining different genera of microorganisms, seeking ease of application with the use of a single inoculant and promoting better plant performance and savings in soybean and common bean crops [4–7].

Co-inoculation in common beans represents a significant technological advance for the crop, especially co-inoculation with *R. tropici* and *Azospirillum brasilense*, which promotes a reduction in production costs and an increase in productivity [4, 5, 7]. Under Cerrado conditions in the central region of Brazil, the average of five field experiments showed that co-inoculation with *R. tropici* and three doses of *A. brasilense* promoted an increase of 100, 74, and 83 kg ha^− 1^, respectively, compared to the use of nitrogen fertilizer, inoculation only with *R. tropici*, and absolute control [4]. Other combinations are gaining prominence, such as the combination of *R. leguminosarum* and *Pseudomonas brassicacearum*, which increased the common bean yield by 15% compared to inoculation with *Rhizobium* alone [8]. In addition to selecting efficient rhizobia and using an appropriate formulation, co-inoculation is an interesting strategy to improve the efficacy of rhizobial inoculants for legumes [8–10].

To increase the use of inoculants consisting of a single bacterium or a combination of bacteria, formulation is the key factor to increasing their efficacy and viability until the moment of application in the field. Industrial-scale production of inoculants involves several factors that are critical to ensuring the quality and efficacy of the final product. Among them, the choice of the vehicle, the consortium of microorganisms, and quality control methodologies are fundamental to guarantee a high number of viable cells and purity of the inoculant and, consequently, its efficiency [11–13]. In this sense, in addition to isolating and evaluating the performance of new combinations of microorganisms that promote better plant development, it is also necessary to research new inoculant formulations, seeking greater efficacy of these products [12].

Based on the above, the aim of this review is to present an overview of the use of inoculants and the history of common bean grain production at a global and national level, focusing on the main mechanisms of action of PGPR, as well as presenting new strategies and technologies to increase the quality and efficiency of inoculants in common bean cultivation.

## Importance and production of common bean

The common bean is a legume grown in various regions of the world, primarily in countries such as Brazil, Myanmar, and India, which are the world’s major producers. The grains of the legume are rich in essential nutrients for human nutrition, such as proteins, carbohydrates, and others, and are considered by many nutritionists to be a complete food [14]. In the 2014–2021 period, the world’s largest bean producers were India, Brazil, Myanmar, China, Tanzania, the United States, and Mexico, producing approximately 5.5, 3.0, 2.9, 1.2, 1.2, 1.2, and 1.1 million tons of beans, respectively [15].

Brazil is the largest producer and consumer of common bean in South America, where the crop is grown under a wide variety of conditions. The crop is grown in different regions, under different soil and climatic conditions, in different production systems (including organic and conventional), with different levels of technology, and on farms ranging in size from small to large. In 2022, approximately 1.6 million hectares of beans were grown in Brazil over the entire crop year, with a total production of 2.5 million metric tons of grain [16]. World common bean grain production and productivity varied widely between 1985 and 2022, as did area. The period between 1997 and 2017 was remarkable for world common bean grain production, standing out as a period of significant increases in area under cultivation and grain production, from 26 million hectares and 16 million tons in 1997 to 37 million hectares and 29 million tons of grain in 2017, resulting in a 30% increase in area under cultivation and a 45% increase in grain production.

However, the average grain yield of common bean is 1571 kg ha^− 1^, which is very low considering the productive potential of the varieties, which exceeds 3000 kg ha^− 1^ [16], besides the fact that in production systems with a high level of technology it is possible to achieve yields above 5000 kg ha^− 1^ [17]. Some factors directly influence the development and productivity of the crop, such as environmental stress, whether due to drought and high temperatures, the occurrence of diseases and low soil fertility conditions [3, 7, 18].

**Fig. 1:**
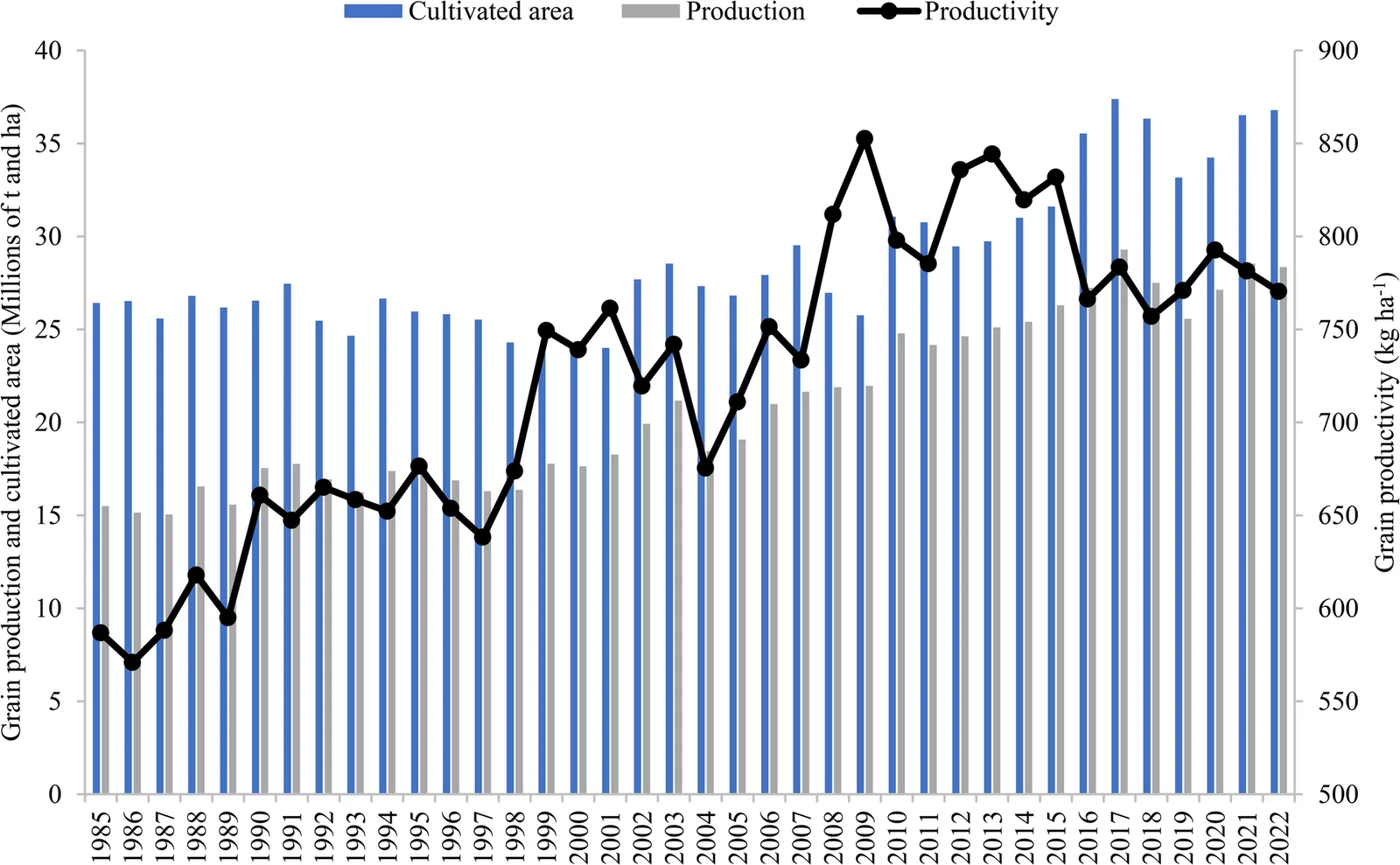
World harvested area, production and productivity of common bean grains from 1985 to 2022. Source: Adapted from FAO [15]

World common bean grain production and productivity varied widely between 1985 and 2022, as did the cultivated area. The period between 1997 and 2017 was remarkable for world common bean grain production, standing out as a period of significant increases in area under cultivation and grain production, from 26 million hectares and 16 million tons in 1997 to 37 million hectares and 29 million tons of grain in 2017, resulting in a 30% increase in area under cultivation and a 45% increase in grain production (Fig. 1).

After this period, between 2017 and 2019, a significant decrease in grain production and planted area was observed by about 4.0 million hectares and 3.6 million tons of grain, respectively (Fig. 1). In the period from 2019 to 2021, the planted area and grain production will increase again by 2.8 million hectares and 2.1 million tons of grain compared to the period from 2017 to 2019 (Fig. 1). With regard to cereal productivity, we can highlight the significant increase of 33% in the period from 1986 to 2009 (Fig. 1). These changes in production and cultivated area, and above all in global grain productivity, demonstrate the importance of implementing new crop technologies in underdeveloped tropical countries.

**Fig. 2:**
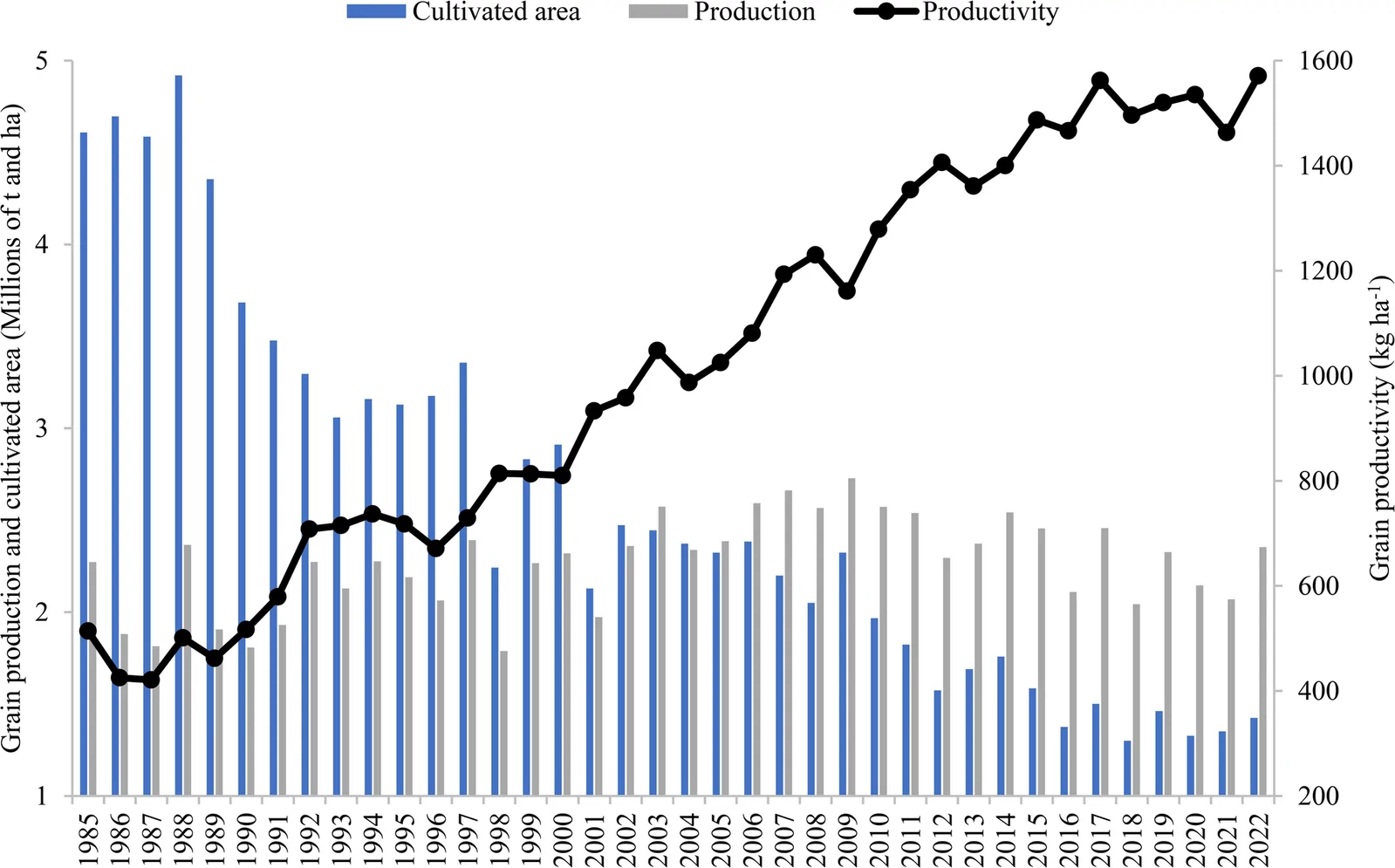
Brazilian harvested area, production and productivity of common bean grains from 1985 to 2022. Source: Adapted from Embrapa Arroz e Feijão [16]

National production and harvested area of common beans also varied widely between 1985 and 2021 (Fig. 2). During this period, grain production varied significantly, especially between 1985 and 2001, as well as between 2010 and 2022. Also noteworthy was the period from 1998 to 2009, when the highest national production occurred, increasing from 2 million tons of grain in 1998 to 2.9 million tons of grain in 2009 (Fig. 2).

Between 1985 and 2022, grain production increased by 3.2%, or about 82,000 tons of grain, while the area under cultivation decreased by 66.2%, from 4.8 to 1.6 million hectares (Fig. 2). The replacement of common bean production areas with commodities such as corn and soybeans, as well as the nutritional preference for other grains such as chickpeas, contributed to this reduction in common bean area. In this context, Mantovani et al. [19], when analyzing the spatial and economic dynamics of bean production in Brazil, highlighted that the volatility of crop prices is related to fluctuations in the supply of the product, which can be conditioned by adverse climatic factors and the increase in production costs, especially due to high fertilizer prices. The increase in costs, associated with the reduction of profitability margins, may discourage producers from growing beans in the subsequent harvest, favoring the replacement of the crop by other agricultural alternatives with greater expectations of economic return. This process can result in a reduction in the area destined to bean cultivation and, consequently, in a decrease in supply, contributing to new fluctuations in prices and evidencing the close relationship between productive and economic factors and decisions to allocate agricultural areas.

Despite the 66.2% reduction in cultivated area, there was a 3.2% increase in cereal production due to the increase in crop productivity, which increased from 514 kg ha^− 1^ in 1985 to 1,571 kg ha^− 1^ in 2022 (Fig. 2). This increase in cereal productivity is due to cultivation on high-tech commercial farms that use more productive varieties, efficient inputs, and state-of-the-art technologies for planting, soil management, and pest and disease control.

In this current context, it is important to implement public policies and new technologies aiming at to increase the cultivated area and productivity of the crop in family farming areas, which account for 70% of common bean production, which could increase national grain production [20–22].

However, the potential contribution of these technologies to increasing common bean productivity should be interpreted with caution, as their effectiveness is strongly influenced by regional, environmental, and socioeconomic conditions. Factors such as the heterogeneous adoption of agricultural technologies among producers, limitations in infrastructure and access to technical assistance, differences in soil and climatic conditions, variability in the performance of inoculants under distinct crop management systems, and constraints related to technology transfer may significantly affect the consistency of the observed benefits. For instance, the efficiency of biological inoculants depends on several factors, including the compatibility between microbial strains and host genotypes, soil physicochemical properties, native microbial communities, environmental conditions, and crop management practices [23–25]. Likewise, the adoption of agricultural innovations in family farming is often constrained by socioeconomic factors, access to information, technical support, and the perceived risks associated with new technologies [26–28]. Therefore, a more comprehensive discussion of these factors would provide a more realistic assessment of the applicability and scalability of these technologies, particularly within family farming systems, where production conditions are highly variable.

## Bacteria of agricultural interest: biological nitrogen fixation for the common bean crop

**Fig. 3:**
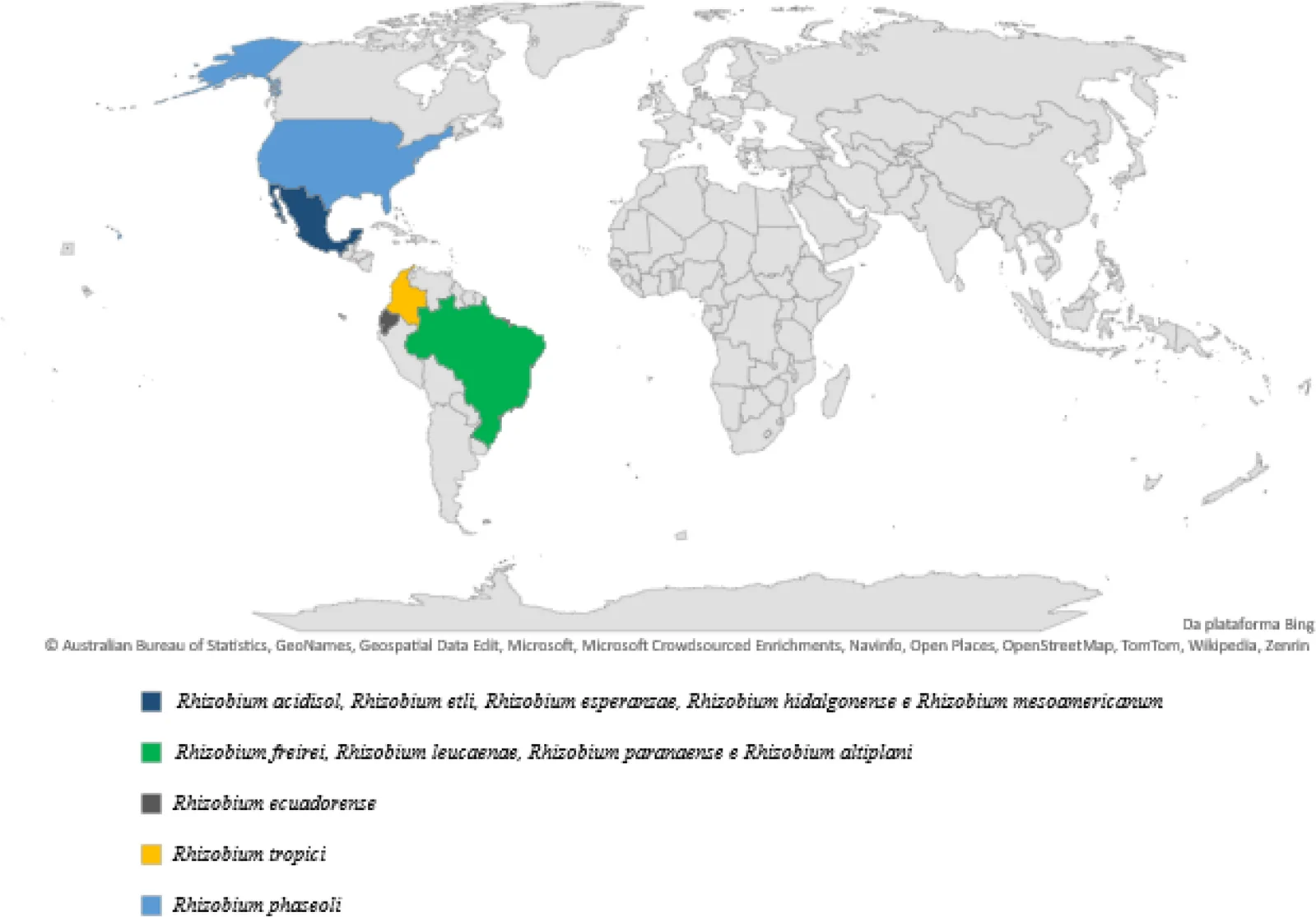
The geographic distribution of the species of isolates *Rhizobium* spp. that nodulated *Phaseolus vulgaris* is in countries of North, Latin and South America by Shamseldin and Velázquez [34] and Barauna et al. [33]

The common bean is considered a promiscuous plant due to its low specificity and can nodulate with different genera and species of bacteria from the alpha-proteobacteria group, such as *Rhizobium*; and beta-proteobacteria, such as *Paraburkholderia nodosa*, *P. tuberum*, *P. sabiae* and *Cupriavidus necator* [3]. Some species of the genus *Rhizobium*, such as *R. tropici* [3, 29], *R. etli* [3, 30], *R. freirei* [3, 31], *R. leucaenae* [3, 32], *R. paranaense* [3, 31] and *R. altiplani* [33] are the most common among those with the ability to nodulate common bean plants (Fig. 3).

The process of selecting efficient N-fixing bacteria includes, in addition to the ability to fix high levels of N, competitiveness, genetic stability, and adaptation to soil and climatic conditions [3, 35, 36]. Following these criteria, currently, there are three bacteria of the genus *Rhizobium* approved in Brazil for the production and commercialization of inoculants for common beans: strains SEMIA 4077 (= CIAT 899) and SEMIA 4088 (= H12) of *R. tropici* and strain SEMIA 4080 (= PRF 81) of *R. freirei* [37]. The strain CIAT 899 has been recognized as an efficient strain in several countries [38, 39].

In a study conducted by Moura et al. [40], the diversity of rhizobia microsymbionts associated with common bean (*Phaseolus vulgaris* L.) was evaluated in the state of Mato Grosso do Sul, Midwest region of Brazil. From phylogenetic, genomic, phenotypic and symbiotic analyses, the authors identified high diversity among the evaluated isolates and proposed five new species of the genus *Rhizobium*: *Rhizobium cerradonense* sp. Nov. (CNPSo 3464^T^), *Rhizobium atlanticum* sp. Nov. (CNPSo 3490^T^), *Rhizobium aureum* sp. Nov. (CNPSo 3968^T^), *Rhizobium pantanalense* sp. Nov. (CNPSo 4039^T^) and *Rhizobium centroccidentale* sp. Nov. (CNPSo 4062^T^). These results show the wide taxonomic diversity of rhizobia associated with common bean in Brazil, highlighting the importance of characterization and prospection of microbial genetic resources for the identification of strains with potential for use in inoculation programs and in the development of biological products for agriculture.

Some biotic and abiotic factors can directly affect the efficiency of the BNF process, making it necessary to supplement the N supply with nitrogen fertilization to ensure high grain yields [41, 42]. Among the main factors are the competition between commercial and native strains present in the soil [43, 44], nodule senescence associated with the cycle of different bean genotypes [45, 46], plant-bacterial interactions [47], and environmental factors such as lack of rain, high temperatures and low soil fertility [48, 49].

In the Cerrado region, the common bean production is still a major challenge due to the dependence on the use of fertilizers, especially nitrogenous ones, which have been used in combination with *Rhizobium* inoculation to increase productivity and reduce production costs [3]. Oliveira et al. [50], when evaluating the inoculation with *Rhizobium tropici* in different common bean cultivars, found that the cultivar BRSMG Uai responded positively to the inoculation with the CIAT 899 strain, presenting productivity similar to that obtained with the application of 80 kg ha⁻¹ of N in the form of urea. In addition, the yield of BRSMG Uai was comparable to that observed in the cultivars BRSMG Madrepérola and Pérola, when inoculated with the same strain, with values close to 3,000 kg ha⁻¹. These results demonstrate the potential of the CIAT 899 strain to promote nitrogen supply to the bean plant through biological N fixation, enabling the achievement of yields similar to those achieved with nitrogen fertilization.

However, promising results have been obtained with the use of biological inputs, as in the study by Messias et al. [2], who conducted five inoculation and co-inoculation trials with common bean in the state of Goiás. The authors reported that inoculation with *R. tropici* alone, without nitrogen fertilization, resulted in an average yield of 3,364 kg ha^− 1^, three times higher than the national yield. This result exceeded that of the treatment with nitrogen fertilization, 3,338 kg ha^− 1^, in which 80 kg N ha^− 1^ was applied. Horácio et al. [51], when evaluating the co-inoculation of cyanobacteria, rhizobia and *Azospirillum* associated with nitrogen fertilization, found that the co-inoculation of common bean, cultivar IPR Curió, with *Anabaena cylindrica*, *Calothrix brevissima*, rhizobia (*Rhizobium tropici* + *R. freirei*) and *Azospirillum brasilense*, associated with the application of 30 kg N ha⁻¹ in topdressing, promoted an increase in grain yield. Regardless of the cultivation time and the N dose applied, the combined inoculation of rhizobia and *C. brevissima* stood out for the increase in grain yield in relation to the non-inoculated control, evidencing the potential of this association as an agronomic practice for the cultivation of common bean under tropical conditions.

Several experiments conducted in commercial common bean production farms have shown that the use of inoculants has demonstrated the possibility of partially, and in some cases completely, replacing nitrogen fertilization with *Rhizobium* inoculation in common bean, with economic and environmental benefits [4, 52]. On the other hand, in the quest to increase the efficiency of inoculation, some strategies have been adopted, such as the use of minimum doses of N in the top dressing, combined with the inoculation of efficient strains [53], as well as the intense search for more efficient N-fixing bacteria [54], with the aim of developing inoculants for common beans.

To ensure high common bean yields through the supply of N via BNF, farmers have adopted practices that increase the efficiency of N fixation, such as applying six doses of *R. tropici* in the sowing furrow. In addition, studies suggest that the application of 15–20 kg N ha^− 1^ together with *R. tropici* inoculation and/or *R. tropici* and *A. brasilense* co-inoculation, at sowing or in the top dressing (stage V4) can improve grain yield [55, 56]. However, care should be taken and it should be noted that applying high doses of N at sowing can reduce nodulation [36, 56].

## Plant development benefits: mechanisms of action of agricultural interest

PGPRs act on plant growth and protection through various mechanisms of action, both direct and indirect. Thus, PGPRs act as biofertilizers, biostimulators, and biocontrollers [57]. Examples of direct action mechanisms include: biological nitrogen fixation, phosphate solubilization, and the production of phytohormones, mainly auxin, gibberellin, cytokinin, and 1-aminocyclopropane-1-carboxylate (ACC) deaminase, an enzyme that reduces ethylene [1, 57].

Indirect action mechanisms include the production of siderophores and also biofilm formation by substances that enhance plant resistance with the production of hydrogen cyanide, bacteriocins, and antibiotics, minimizing the action of phytopathogens [57–59]. They also have the ability to secrete volatile metabolites, produce exopolysaccharides, osmoregulators, and antioxidants that help plants protect themselves from disease and tolerate abiotic stress [1, 57].

## Biofertilizers

Biofertilizing PGPRs or biofertilizers are so named because they provide or increase the uptake of nutrients essential for plant development, promote the maintenance of physiological processes, improve plant performance, and reduce the need for synthetic fertilizers [57]. According to Cortivo et al. [60] and Lopes et al. [57], microorganisms are usually selected as nitrogen fixers, phosphate solubilizers, and siderophore producers because of their potential as biofertilizers.

N is part of several biomolecules, such as proteins and nucleic acids, making it the most important nutrient for plants. The use of N-fixing bacteria, such as bacteria from the genera *Bradyrhizobium*, *Azospirillum*, *Azotobacter*, *Achromobacter*, *Beijerinckia*, *Rhizobium*, and *Frankia*, which fix atmospheric nitrogen (N_2_) and reduce to ammonia (NH_3_), making it available to plants [1, 57, 58, 61], is a viable alternative for replacing or reducing the use of nitrogen fertilizers.

Phosphorus (P) is the second most important nutrient required by plants and, like N, plays a fundamental role in the physiological performance of plants as it is part of important molecules such as phospholipids and adenosine triphosphate (ATP), which play a key role in photosynthesis [57]. However, much of the P present in the soil is in non-labile form, i.e., unavailable to plants, which affects their performance. Some PGPR’s have the ability to solubilize phosphate, making it available to the plant by converting insoluble phosphate into soluble ions [57, 62].

According to Lopes et al. [57] and Umesha et al. [63], bacteria from the genera *Agrobacterium*, *Bacillus*, *Pseudomonas*, *Rhizobium*, *Achromobacter* and *Burkholderia* solubilize inorganic phosphorus and convert it to monobasic phosphate (H_2_PO^− 4^) or dibasic phosphate (HPO_4_^−2^), making it available for uptake by plants. Another mechanism used by these microorganisms to solubilize phosphate is the change in pH [1, 57]. In alkaline soils, microorganisms secrete organic acids such as gluconate, succinate, lactate, and citrate, which act to solubilize calcium phosphate (Ca_3_(PO_4_)_2_) by lowering the pH of the soil [1, 57]. On the other hand, in acidic soils, microorganisms increase soil pH by producing protons during ammonium (NH4^+^) assimilation, solubilizing aluminum phosphate (AlPO_4_) and ferric phosphate III (FePO_4_), and improving plant adaptation and development [57].

Another nutrient necessary for the physiological maintenance of plants is iron (Fe), which is associated with chlorophyll biosynthesis, photosynthesis, and plant respiration [57]. Microorganisms of the genera *Azotobacter*, *Bacillus*, *Pantoea*, *Rhizobium*, *Pseudomonas*, *Burkholderia*, *Enterobacter*, *Klebsiella* and *Azospirillum* that can produce catechol, hydroxamate and carboxylate type siderophores are classified as Fe binding and Fe chelating [64, 65]. According to Sun et al. [65], the microorganism detects the low concentration of iron and then the siderophore is secreted to immediately chelate iron (III) in the form of a siderophore-Fe^3+^ complex, which is recognized and translocated into the cell. In the cell cytosol, the siderophore-Fe^3+^ complex undergoes a reduction reaction and the iron is released in ferrous form. In addition to improving plant nutrition, siderophores help inhibit phytopathogens and degrade pesticides in the environment by sequestering iron from the soil [65].

Other essential nutrients for plant development are sulfur (S) and potassium (K). S is associated with the composition of the amino acids cysteine and methionine [61], and K is directly associated with plant physiological performance, respiration, and nutrient transport [61]. Bacteria of the genus *Bacillus*, which produce dimethyl disulfide, make S available to plants. Bacteria of the genera *Bacillus*, *Burkholderia*, *Arthrobacter*, *Pseudomonas*, *Enterobacter*, *Agrobacterium*, and *Rhizobium* are capable of solubilizing K through the production of organic and inorganic acids, proton production (acidolysis mechanism), chelation, and exchange reactions capable of converting insoluble K (mica, muscovite, and biotite feldspar) into soluble forms of K that are easily absorbed by the plant [66].

## Biostimulators

PGPRs can also act as biostimulators by synthesizing plant growth regulatory molecules that mimic phytohormones, improving development and optimizing the physiological response of plants to adapt to adverse stress conditions [1, 57, 67, 68]. Some examples of bacterial genera that act as biostimulants are *Azospirillum sp*., *Pseudomonas sp*. and *Bacillus sp*. They are capable of producing auxin [69], cytokinin, gibberellin, ethylene (ACC deaminase), abscisic acid, jasmonates and strigolactones [1, 67, 68].

Auxin is essential for plant growth as it is involved in cell division of vascular tissues and root development of plants, allowing the expansion and production of new tissues [67, 68]. Another beneficial phytohormone for plant root development is cytokinin, which acts on cambial activity, cell differentiation, and apical dominance [1, 68], facilitating the colonization of N-fixing bacteria such as *Rhizobium*. Gibberellin is important for improving the germination process, the development of the aerial part and acts in the reproductive phase of plants, in the increase and development of the number of flower buds and in leaf senescence [1, 57, 68].

PGPR’s act by increasing the concentration of ACC deaminase, which helps to reduce ethylene, thereby mitigating the effects of abiotic stress on plants. Controlling ethylene levels is essential because high levels are detrimental to plants, reducing the development of the plant’s aerial part and roots, leading to a reduction in grain production [1, 67, 68]. Another way in which these microorganisms affect the physiological performance of plants is by increasing the concentration of abscisic acid, which causes stomatal closure and reduces water loss, as well as jasmonates and brassinosteroids, which can increase the concentration of calcium (Ca^2+^) in plants [70].

## Biocontrollers

Another way PGPR’s can act is to protect plants as biocontrol agents or by producing substances that help control diseases, pests and pathogens. Some microorganisms belonging to the genera *Agrobacterium sp*., *Bacillus sp*. and *Pseudomonas sp*. can act as bactericides, while microorganisms belonging to the genera *Pseudomonas*, *Streptomyces* and *Burkholderia* can have a fungicidal effect.

In terms of insecticidal activity, the genera *Bacillus*, *Clostridium*, *Pseudomonas*, *Saccharopolyspora* and *Streptomyces* stand out [71], while the genera *Lysobacter*, *Pseudomonas* and *Bacillus* have nematicidal activity [72]. According to Glick [1], PGPR’s promote the induction of systemic acquired resistance (SAR) and induced systemic resistance (ISR) through the production of phytoalexins, exopolysaccharides (EPS) and antioxidants. SAR is a salicylic acid-dependent, nonspecific system that acts against biotrophic pathogens. In contrast, ISR is triggered by jasmonic acid and is activated by pathogenic microorganisms [73].

For the control of pathogens in the processes of infection, development and reproduction of pathogens, bacteria of the genus *Pseudomonas spp*. can have a direct antagonistic effect on soil pathogens and causes of disease in the aerial part [57]. This effect occurs through the production of antioxidants such as catalase (CAT), superoxide dismutase (SOD), ascorbate peroxidase (AsA), glutathione (GSH), carotenoids, tocopherols and phenolic compounds [57, 70, 74].

The formation of biofilms produced by bacteria of the genus *Bacillus spp*. is also a form of control, as they are composed of EPS that protect the root system from external stresses, reduce competition with the microbiota, and increase antimicrobial activity in the soil [68]. Another tool is secondary metabolites that some microorganisms can produce, some examples are the genera *Pseudomonas* spp. and *Bacillus* spp. that produce antibiotics and enzymes such as chitinases, glucanases, and peroxidases [75].

## Registration and commercialization of inoculants in Brazil

The success of inoculation technology is the result of more than one hundred years of research. The first studies to develop the first inoculants were based on rhizobium cultures, which were sold in the United States from 1896 [76]. Currently, products composed of commercial strains are sold in various parts of the world. Nowadays, there are 65 *Rhizobium*-based inoculants and 5 *Azospirillum*-based inoculants, for a total of 70 inoculants registered for common beans in Brazil [77]. While, in the application of the National Bioinput Program (PNB) for bean cultivation there are already 76 inoculants based on *Rhizobium*, 7 inoculants based on *Azospirillum*, 2 inoculants based on *Bacillus* and 2 inoculants based on *Pantoea*, totaling 87 registered inoculants [78]. These inoculants are sold in liquid and/or solid physical nature (peat). Regarding the concentration of these bacteria, inoculants containing *Rhizobium*, the concentration varies from 1.0 × 10^9^ to 4.0 × 10^9^ UFC mL^− 1^ or g^− 1^, inoculants containing *Azospirillum*, the concentration varies from 2.0 × 10^8^ to 5.0 × 10^8^ UFC mL^− 1^, inoculants containing *Bacillus* concentration ranges from 4.0 × 10^9^ to 2.5 × 10^10^ UFC mL^− 1^ and the inoculants containing *Pantoea* at a concentration of 2.0 × 10^9^ UFC g^− 1^, respectively [78].

With the significant results obtained in recent years regarding the efficiency on the use of microorganisms, both in the application of rhizobia and in the use of PGPR’s, an increase in the number of inoculants registered for common bean is expected. According to Brazilian legislation, in order to register an inoculant, feasibility and agronomic efficiency tests must be carried out using protocols established by the Ministry of Agriculture and Livestock (MAPA) through MAPA/SDA Normative Instruction No. 13 of 2011 [37]. In addition to registration, it is necessary to follow a set of requirements for the quality control of inoculants, established by MAPA/SDA Normative Instruction No. 30 of 2010 [79]. It is worth noting that regarding rhizobial inoculants, the legislation also establishes a minimum concentration of 1 × 10^9^ viable cells/g or mL until the expiration date, which must be at least 6 months, and free of contaminants at a dilution of 1 × 10^− 5^ [37]. The technical recommendation in Brazil indicates a dose that allows for at least 1.2 million viable cells per seed to ensure successful nodulation [80]. According to Santos et al. [56], the credibility of the inoculant market in Brazil depends on strict legal regulation.

**Fig. 4:**
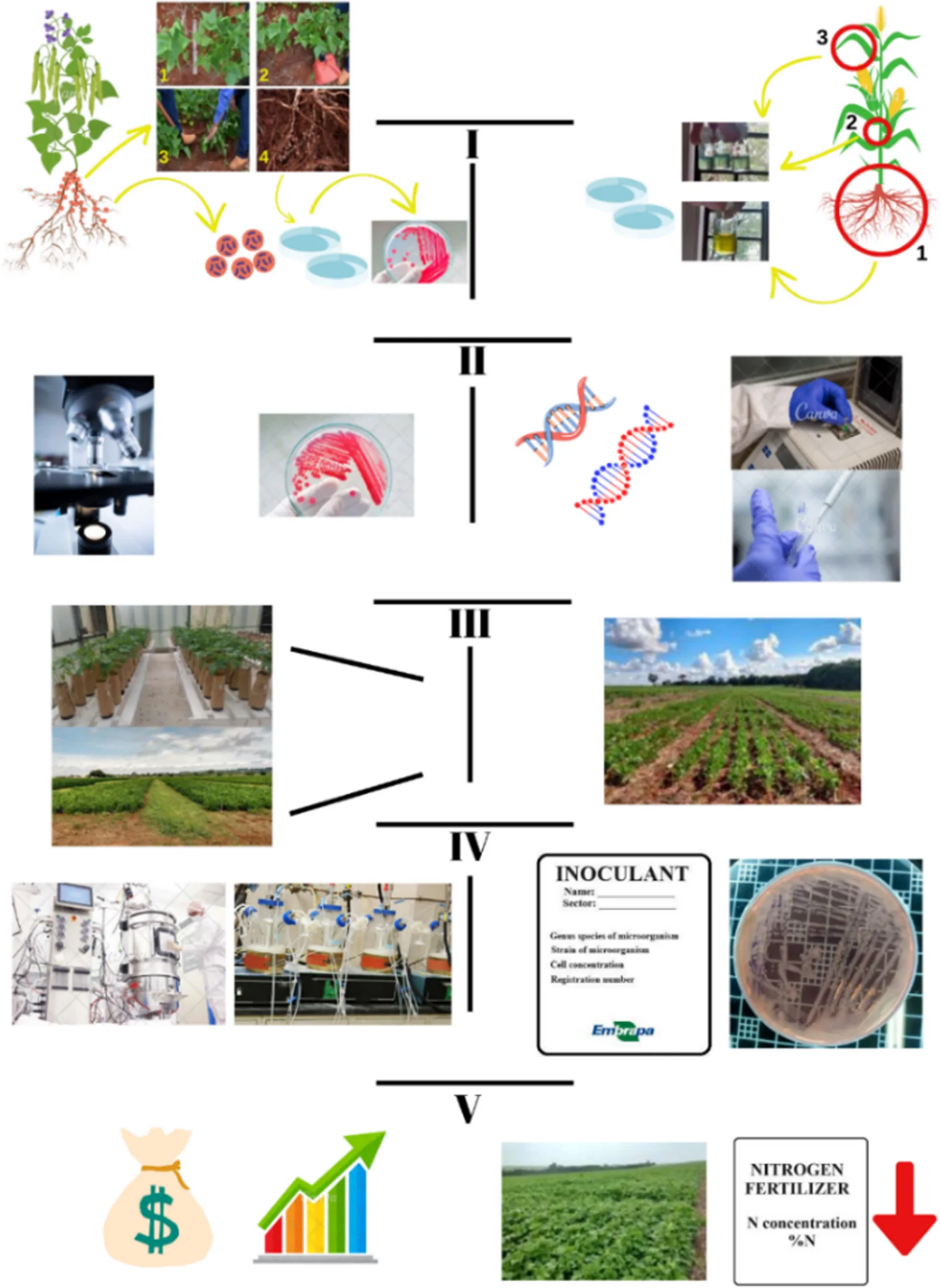
Development process and benefits of using inoculants according to Embrapa. I- Collection of microorganisms from legumes and grasses, II- Isolation, identification and characterization of microorganisms, III- Agronomic efficiency testing, IV- Product development and application, and V- Economic benefits and reduction of synthetic fertilizers and chemical insecticides and fungicides. Source: Embrapa Agrobiology and the Ministry of Agriculture and Livestock (MAPA) through Normative Instruction MAPA/SDA No. 13 of 2011 [37]. (adapted)

Usually, the process of developing a product follows some steps of the MAPA [37], which are as follows 1- Collection of microorganisms from legume and grass plant species; 2- Isolation, identification and characterization of microorganisms through DNA sequencing, genes of interest, biochemical tests and morphological characterization; 3- Agronomic efficiency testing: Tests in test tubes, sterilized pots and, with non-sterile soil and field trials; 4- Development, production and application and product quality and; 5- Economic benefits and reduction in the use of synthetic fertilizers with the use of inoculants containing N-fixing bacteria, P and K solubilizers and, fungicides and chemical insecticides with the use of bacteria producing toxic substances to control fungi and insects (Fig. 4).

The use and development of multifunctional microorganisms in agriculture have increased considerably in recent years, particularly in soybean and common bean production systems, owing to their potential to promote biological nitrogen fixation, nutrient acquisition, plant growth, and disease suppression. However, it is important to distinguish between multifunctional microorganisms and multifunctional inoculant formulations. Although several commercial microbial products are currently registered in Brazil, most inoculants are formulated with a single microbial strain or species. These microorganisms may exhibit multiple plant growth-promoting mechanisms or be used in co-inoculation strategies, in which two or more inoculants containing different microorganisms are applied simultaneously at sowing. Thus, co-inoculation represents the main approach currently adopted in both research and commercial production to combine the complementary functions of different beneficial microorganisms, rather than relying on inoculant formulations containing multiple microbial strains in a single product [81–85].

The use and search for multifunctional microorganisms in agriculture has been growing in crops such as soybeans and common beans. However, there is currently only one inoculant with more than one microorganism registered in Brazil for soybean crops, the Innova Agrotecnologia’s COMBIO, composed by the microorganisms *Bradyrhizobium elkanii* (BR 29), *Bacillus subtilis* (BR 10788) and *Paraburkholderia nodosa* (BR 10141). Research is advancing with inoculants that have more than one mechanism of action for corn and common bean crops, with microorganisms that act as biofertilizers, biostimulants and, above all, biocontrollers. It should be noted that between 2015 and 2018, there was a 220% increase in the commercialization of inoculants composed of *Azospirillum brasilense* [86]. However, this increase in the commercialization of *A. brasilense* inoculants is due to the co-inoculation of inoculants recommended for legumes [56], which are typically recommended for grasses such as wheat and corn. Data showing the success of co-inoculation of rhizobia and *A. brasilense* with strains AbV5 and AbV6 demonstrate the success of this technology, which is now registered for common bean in Brazil and is a reality for most farmers. This encourages further research into the combination and application of different beneficial microorganisms to bean crops.

In this topic, multifunctional microorganisms stand out because they have more than one mechanism of action, such as N fixation, stimulation and production of phytohormones, and as biocontrol agents [12, 87, 88]. One of the barriers to common bean incorporation is mainly rhizobia with low competitiveness and low BNF efficiency. Another point is the production of inoculants with consortia of microorganisms. However, there are some factors that pose a risk to the multifunctional inoculant production system, such as contamination by hazardous microorganisms, a common source of carbon, compatibility between the microorganisms themselves, and the type and quality of the vehicle carrying the microorganisms.

These are some of the factors that affect the commercialization and efficiency of this type of inoculant in the field, which it would be important to standardize and avoid inconsistent experimental results, in addition to identifying and controlling harmful microorganisms in the production process, guaranteeing the quality of the inoculant [89]. In addition to these factors, the on-farm production system is currently gaining prominence in Brazil, but it is a complex system that, due to the difficulty of controlling and containing a large number of contaminants, leads producers to produce and apply low-efficiency inoculants to their crops.

**Fig. 5:**
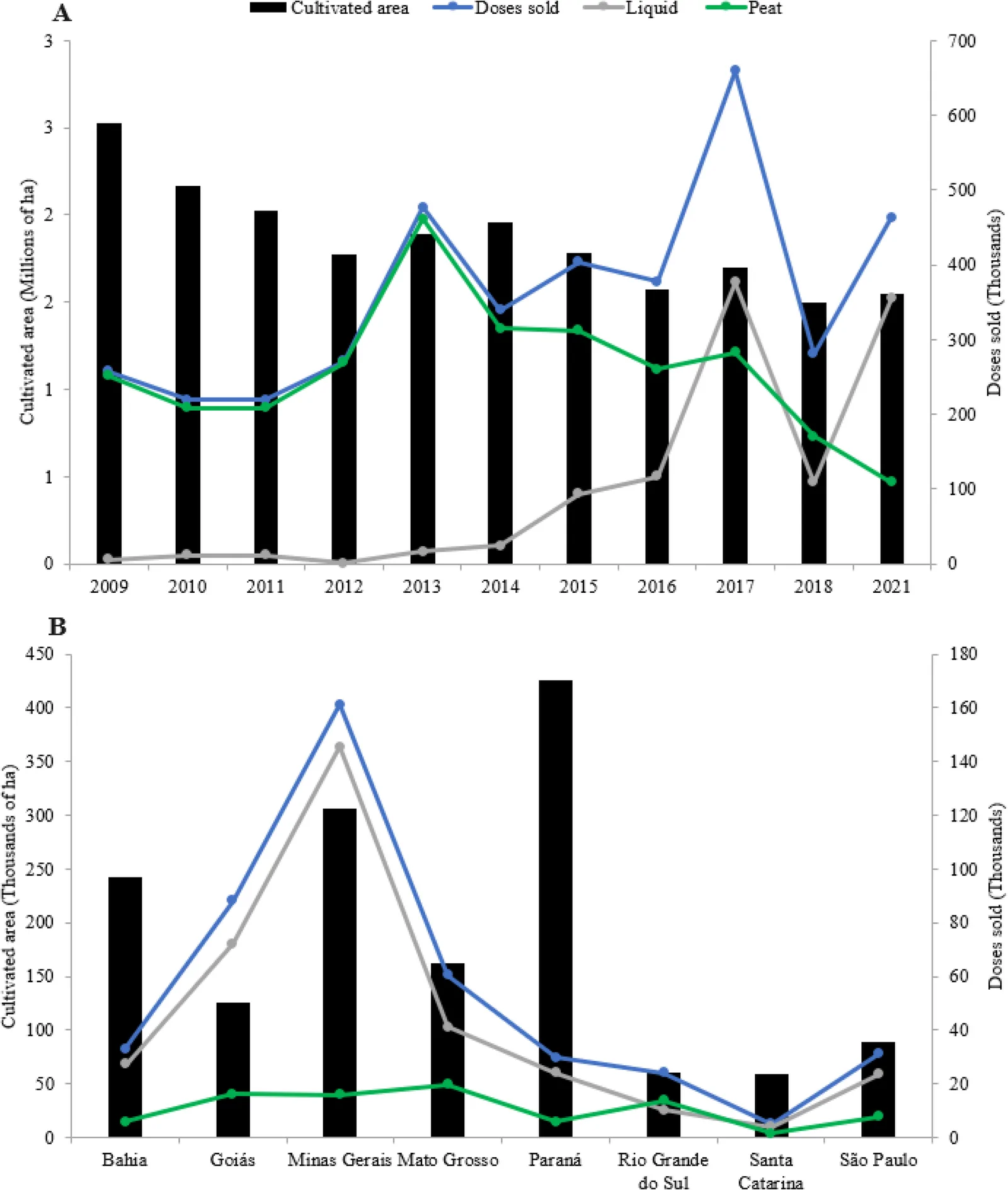
Total commercialized doses of liquid and peat inoculants associated with the cultivated area of common beans in the period 2009–2021 (5 **A**). Cultivated area and total commercialized doses of liquid and peat inoculants for common beans associated with the main Brazilian states where inoculants are used in 2021 (5**B**). Source: ANPII [86] and Embrapa Arroz e Feijão [16]

From a marketing point of view, the type and condition of inoculants contribute to the ease of application and efficiency in the field, which shows the preference in recent years for the use of peat inoculants in bean production, reaching approximately 131% of the total used in 2021 [86]. Between 2009 and 2013, the number of doses marketed increased from 257,398 to 477,848 (Fig. 5A) [86]. After this period, between 2013 and 2016, there was a decrease from 200,871 to 100,285 doses of peat inoculants marketed. During this period, there was a clear preference for the use of liquid inoculants among Brazilian common bean growers, with an increase of 100,856 doses sold (Fig. 5A).

In 2017, a total of 658,503 doses were sold, of which 376,760 were liquid inoculants. Despite the decrease in the number of doses sold in 2018, there was an increase in the total number of doses and doses of liquid inoculants in 2021, with 463,015 and 353,958 doses sold, respectively (Fig. 5A). This variation is related to the predominance of common bean growers who have used nitrogen fertilizer, as well as the quality and efficiency of the inoculants. The reduction and variation in the use of inoculants is also associated with the reduction in the area under common bean cultivation, from 2.5 million hectares in 2009 to 1.6 million hectares in 2021, representing a 36% reduction in the area under cultivation (Fig. 5B) [16].

In 2021, the three leading Brazilian states in terms of total doses sold and use of liquid inoculant doses were Minas Gerais, Goiás and Mato Grosso. The number of doses marketed in these states was 161,218, 88,189 and 60,507, respectively, while the number of liquid inoculant doses was 145,482, 72,018 and 41,172, respectively (Fig. 5B) [86]. In the same year, 20,016 doses of peat inoculants were exported. The three states with the highest number of doses marketed, Minas Gerais, Mato Grosso and Goiás, also have the largest areas under bean cultivation, with 306,560, 161,962 and 125,326 ha, respectively, while the state of Paraná, which has one of the lowest numbers of doses marketed, has a cultivated area of 426,400 ha (Fig. 5B) [16].

The low number of commercialized doses of inoculants in Brazil for the common bean crop seems to be related to factors of agronomic, economic and technological adoption by producers. Although the use of inoculants containing N-fixing bacteria is highly valued for reducing dependence on synthetic fertilizers, its adoption still faces challenges, especially in the common bean crop. Challenges include a lack of technical knowledge and training from companies to farmers on the benefits of inoculation, as well as competition from nitrogen fertilizers, which provide immediate results but are less sustainable. Another issue is the logistical problems of distribution and storage conditions that require the availability and efficacy of these products. In addition, fluctuations in demand, influenced by factors such as bean acreage and fluctuations in bean prices, have a direct impact on the inoculant market, which may contribute to the observed decline in sales of inoculant doses for this crop. Therefore, in order to reverse this situation, it is necessary to invest in rural extension programs.

From an economic point of view, the increase in the price of common bean from 2009 to 2021 reached an average value of approximately R$ 161.00 per bag. However, in the period from 2022 to 2024, the average value of the bag reached R$: 303.00, representing an increase of 53% compared to the period from 2009 to 2021. This increase in the price of grains on the market allows producers to invest more in nitrogen fertilizers, which contributes to a reduction in the sale of inoculant doses for common beans.

## Main inoculant formulations: advantages and disadvantages

Commercial inoculant formulations are liquid or solid, including powdered or granular vehicles, liquids, pellets, and especially peat vehicles. Currently, the majority of doses marketed are in liquid formulations (Fig. 5) [86]. Formulation is one of the key factors in the efficiency and profitability of commercial inoculants [12, 56, 90]. According to Bashan [11] and Bashan et al. [12], the inoculant formulation process can determine the direction between the commercial success or failure of the biological agent, so chemical and physical properties, manufacturing and farm management qualities, a friendly environment and storage quality are fundamental to producing a quality formulation. Some examples of inoculant formulations were investigated by Bashan et al. [12] and are shown in Table 1.

**Table 1:** Examples of liquid and solid formulations used to produce plant growth-promoting bacteria inoculants from 1998 to 2014

Type of formulation	Type of inoculant	Aditives or treatments	Used microrganisms
Liquid	-	Glycerol, PVP, trehalose and FeEDTA	Bradyrhizobium japonicum
		PVP and FeEDTA	Bradyrhizobium japonicum and Bacillus megaterium
		Sucrose	Bacillus subtilis, B. amyloliquefaciens and Pseudomonas spp.
		Arabic gum	Bradyrhizobium sp., Rhodobacter capsulatus and Rhizobium sp.
		Glycerol	Pseudomonas fluorescens
Solid	Peat	None or with undisclosed additives. Applications such as: seed and pellet coating	Azospirillum brasilense, A. Lipoferum, Bradyrhizobium japonicum, Rhizobium sp. and Rhizobium leguminosarum
		Charcoal and adhesive	Klebsiella pneumonia, Pantoea agglomerans, Gluconacetobacter diazotrophicus and Herbaspirillum seropedicae
		Sugar	Azospirillum brasilense
		Arabic gum	Rhizobium spp. and Bradyrhizobium
	Coconut powder/coconut peat	None	Azorhizobium caulinodans
	Wheat or oat bran	CMC	Bacillus subtilis and Pseudomonas putida
	Grape pomace and cork compound	Arabic gum and Carboxymethyl cellulose	Various rhizobia, Bradyrhizobium japonicum and Bacillus megaterium
	Clay and perlite minerals	Arabic gum and CMC	Various rhizobia; Bradyrhizobium japonicum and Bacillus megaterium
	Talc	CMC	Bacillus subtilis and Pseudomonas putida
	Perlite	Arabic gum	Rhizobium and Bradyrhizobium
	Alginate	None	Azospirillum brasilense, A. lipoferum, Pseudomonas fluroescens and Bacillus megaterium
	Alginate	None	Rhizobium spp.
	Alginate	Humic acid	Pseudomonas putida and Bacillus subtilis
	Alginate	Skimmed milk	Bacillus subtilis and Pseudomonas corrugata
	CMC/cornstarch	MgO	Rhizobia, Gluconacetobacter diazotropicus, Herbaspirillum seropedicae, H. rubrisubalbicans, Azospirillum amazonense and Burkholderia tropica

*CMC* Carboxymethylcellulose, *MgO* Magnesium oxide, *FeEDTA* Iron chelate, *PVP* Polyvinylpyrrolidone. Fonte: adaptado de Bashan et al. [12]

Bashan [11] and Bashan et al. [12] emphasize the importance of chemical and physical properties, which affect sterilization processes and water retention capacity. In terms of manufacturing quality characteristics, thinking about ease in the manufacturing process, they should allow the addition of specific nutrients and essences, have an easily adjustable pH, and be made from low-cost raw materials. These characteristics are essential to ensure increased cell viability of the formulations and to support as many bacterial species as possible.

As for agricultural handling quality, it is essential at the time of inoculant application to allow for more practical handling and controlled release through standard planting machines. In terms of storage quality, it must meet the minimum guarantee of viable cell concentration for a sufficient period of time, associated with minimum and maximum temperature conditions and the types of environments recommended for storage. Finally, one of the most important characteristics of the formulations is that they should be environmentally friendly, i.e. non-toxic, biodegradable, leave no carbon footprint and be non-polluting.

It is worth noting that in addition to the characteristics, there are some factors that can influence the quality and efficiency of inoculants. One of them is the time of mixing the bacterial culture (logarithmic or stationary phase) and the type of vehicle, which directly affects the concentration of active cells [11, 12]. In addition, it influences the forms of inoculation, recommended dosage associated with the use of technologies for applying the inoculant [12].

## Liquid inoculants

Liquid formulation inoculants are microbial cultures combined with various modified compounds, such as water, oil or polymeric substances, which aim to increase adhesion, stability, viscosity, surfactant capacity and dispersion [12, 90, 91]. Typically, this type of formulation consists of 10 to 40% microbial culture, 1 to 3% suspending ingredient, 1 to 5% dispersant, 3 to 8% surfactant, and 35 to 65% carrier vehicle [92, 93].

In this type of formulation, the use of additives is essential for the maintenance and survival of microbial cells, such as sucrose, which improves the survival of rhizobia. The addition of glycerol not only helps to preserve the cells [12], but also acts as a corrective, retaining the necessary amount of water and protecting against desiccation, thus reducing the drying rate [12]. Carboxymethylcellulose (CMC) is a common and important additive due to its water-soluble properties and relative low cost. Arabic gum arabic (AG), on the other hand, is a carbohydrate used as an adhesive [12, 13], which aims to protect bacterial cells from desiccation and may also improve the viability of inoculants by maintaining a greater number of active cells. This type of carbohydrate is typically used for inoculants containing rhizobia.

AG, xanthan gum (XG), gelatin and alginate are examples of natural polymers that act as protection agents. Polyvinyl alcohol and polyvinyl pyrrolidone (PVP), on the other hand, are synthetic polymers that, in addition to vegetable oil and glycerol, are suitable for the development of liquid formulations [12, 90]. These protective agents are associated with providing nutrients and improving the physical properties and osmoprotection of bacterial cells [12, 90, 91]. In addition, these compounds help bacteria survive extreme osmotic stress. According to Lobo et al. [90], natural and synthetic polymers provide a protective microenvironment for bacterial cells that can limit heat transfer and have high water activity (aw). These polymers promote greater bacterial cell survival under various storage conditions [90].

These liquid inoculants have some advantages over solid inoculants, such as greater convenience and ease of application, providing better seed coverage. In addition, this type of inoculant can be used in combination with other technologies and, most importantly, has a low-cost production process. Lobo et al. [90] emphasize that liquid formulations allow the manufacturer to include sufficient amounts of nutrients, cell protectors, and inducers responsible for cell formation to improve effective performance in the field. Usually, liquid inoculants have a high cell concentration, which allows the use of reduced doses of the inoculant [92]. Regarding the method of application in the field, spraying not only makes it easier for the grower, but can also ensure better distribution of the inoculant on the seed surface.

On the other hand, the disadvantage of this type of formulation is the limited shelf life due to the low number of viable cells in the inoculant caused by nutrient depletion, heat shock, or hypoxia [90]. Therefore, it is essential to have a cool or refrigerated environment for inoculant storage [90]. To overcome these issues and increase the efficiency of these inoculants, it is necessary to improve and/or adapt the formulations [12, 90, 91].

## Solid inoculants

Inoculants for solid formulations can be developed in organic or inorganic vehicles and can be produced in granular or powder form. These types of formulations have good stability of viable cells during storage over a long period of time. In addition, they are classified based on their characteristics, such as particle size or mode of application [12, 56, 90, 91].

Wet solid formulations are typically developed in alginate [94], clay [95], peat [96], and biochar [97]. These types of formulations make it possible to prolong the survival of microbial cells under high-temperature conditions [98]. In this context, as in the case of liquid inoculants, the vehicles must be low-cost, have high availability, chemical stability and low toxicity, as well as flexibility in application for farmers [12, 99].

The specificity of dry formulations is that they need to be rehydrated depending on the application method to achieve greater seed coverage [99]. This type of inoculant is produced using processes such as air drying, desiccation, freeze drying and spray drying. The choice of production method depends on the cost-benefit ratio on an industrial scale. Among the above mentioned processes, lyophilization is the most widely used in the production of this type of inoculant as it produces the cell-protectant mixture, forms a protective matrix and maintains the survival of the microbial cells [90, 100].

In powder formulations, talc is used as a bacterial carrier. According to Martínez-Álvarez et al. [101], talc has a corresponding hydrophobicity and reduced moisture absorption, which, in addition to preventing the formation of hydrate bridges, favors long-term storage. As in other types of formulations, carrier vehicles are combined with adhesive and protective agents such as CMC [90, 101], gelatin [90], AG [90, 102], maltodextrin and sucrose [90]. The use of these protectants is essential for the survival and maintenance of cells during drying and storage [95]. In this context, an example of a stabilizer and preservative is sodium glutamate, which can increase the fluidity of the cell membrane and stabilize the head group, promoting membrane preservation and increasing bacterial resistance to the drying process [90].

The use of cell protectants is essential to preserve bacterial viability during drying and storage by minimizing membrane damage, protein denaturation, and oxidative stress [56, 103, 104]. Among the compounds investigated, monosodium glutamate has been widely used as an osmoprotectant and membrane stabilizer during spray- and freeze-drying processes. Experimental studies have shown that monosodium glutamate, particularly when combined with sugars such as sucrose or maltodextrin, significantly improves bacterial survival by preserving membrane integrity and reducing dehydration-induced cellular damage [12, 45, 105, 106]. In addition, other protectants, including polyvinylpyrrolidone (PVP), glycerol, trehalose, sodium alginate, and carboxymethyl cellulose, have demonstrated the ability to enhance bacterial survival during formulation and storage by maintaining cellular hydration and improving resistance to environmental stress [12, 56, 104, 107].

Among solid formulations, the preferred carrier for rhizobia is peat, also known as peat inoculants, and it is the most widely used carrier in North and South America, Europe, and Australia [12]. Peat is a solid organic carrier formed by a natural process in the soil over a long geological period [56]. The choice of this type of carrier has some advantages due to its high organic matter content and water retention capacity, which are important characteristics to promote the proliferation of bacterial cells over a period of time [56]. In addition, its matrix promotes greater protection and survival of bacterial cells under conditions of water restriction and high temperatures, especially when sterilized by gamma radiation or autoclaving [12, 103].

For the production of peat-based inoculants, a number of characteristics must be considered, such as its granulometry, pH, ideal humidity and quality control [12, 103]. Furthermore, due to its high demand, there is an increase in the exploitation of peatlands, which can lead to some environmental impacts, such as habitat destruction and CO_2_ emissions [56]. Due to the scarcity of peatlands in Brazil, it is necessary to import peat, which increases the cost of the production process of this type of inoculant [56].

Although peat inoculants are widely recommended for rhizobia due to their high cell stability, they have some disadvantages when it comes to production and application in the field. Finding peat with an ideal pH (between 3.5 and 8.5), good water retention and high cation exchange capacity [12] is a challenge that can increase production costs. In addition, there is the need for sterilization and pH correction with calcium or magnesium carbonate [12, 103], which, in addition to the other characteristics mentioned above, can further increase the cost of the production process. In the field, the application of peat inoculants requires specific techniques to ensure a more uniform distribution on the seeds, which can also be an operational obstacle to the use of this type of inoculant.

## Benefits and challenges of co-inoculation for the common bean production system

The use of beneficial bacteria in Brazilian agriculture has brought economic and environmental benefits, especially in soybean and common bean production systems. In recent years, the development of inoculants containing different strains and/or bacterial species with different mechanisms of action has increased significantly. These inoculants aim to improve plant development, enhance protection against biotic and abiotic stresses and, above all, increase grain yield. In this sense, the use of these inoculants has expanded to several crops, consolidating itself as a strategic technology for sustainable agriculture.

**Fig. 6:**
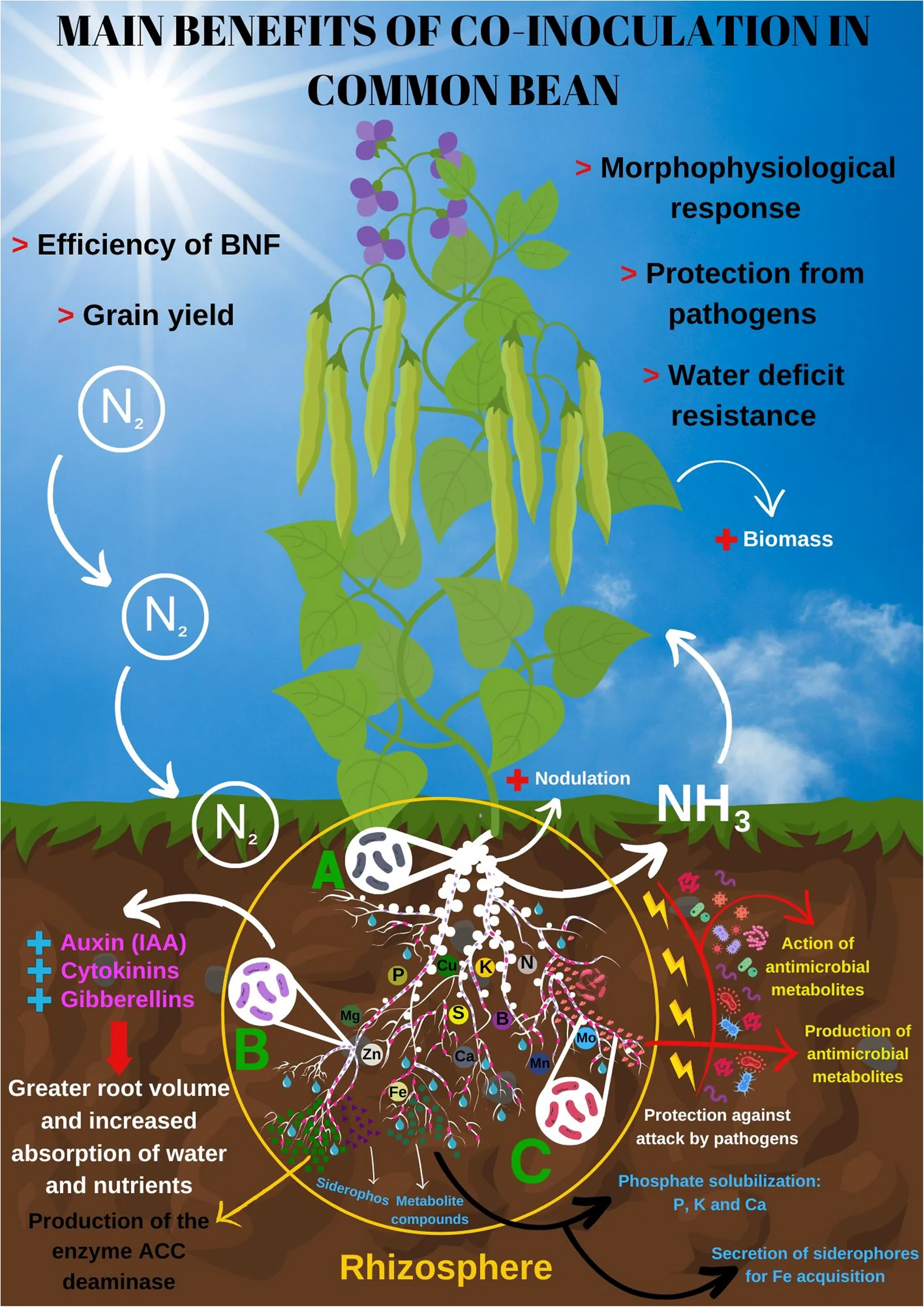
Benefits of co-inoculating rhizobia with growth-promoting bacteria in common bean plants, acting as biofertilizers (A), biostimulants (B) and biocontrollers (C). This figure was adapted from Barbosa et al. [111] created by placing the benefits of co-inoculation in common bean

These inoculants are known as “mixed inoculants” or “multifunctional inoculants,” and the practice is commonly referred to as co-inoculation, which involves the application of two microorganisms. However, despite the economic and environmental benefits of these inoculants, there are still issues to consider that may affect the efficiency of co-inoculation. This efficiency is closely related to factors such as strain selection and compatibility, cell concentration, dosage, product type, inoculation method (e.g., seed application, foliar or in-furrow spraying), and plant genotype [56]. Thus, the development of new studies focused on the formulation of commercial inoculants with different bacteria [56, 108] and alternative application methods [56, 109, 110] has been steadily increasing. The combination of microorganisms has proven to be an effective strategy for ensuring high grain yields in bean production in Brazil. In addition, this combination promotes beneficial effects by increasing symbiotic efficiency, nodulation, plant development, as well as providing greater protection against phytopathogens and tolerance to abiotic stresses (Fig. 6).

The synergistic interaction between *Rhizobium* spp. and *Azospirillum brasilense* has been extensively documented in both soybean and common bean. *Azospirillum* promotes the synthesis of phytohormones, particularly indole-3-acetic acid (IAA), gibberellins, and cytokinins, stimulating lateral root development and increasing root hair density. These changes enlarge the root surface area and create additional infection sites for rhizobial colonization, resulting in greater nodule number and biomass, enhanced nitrogenase activity, and increased biological nitrogen fixation [23, 112]. In a review of more than 400 field experiments, Hungria et al. [82] reported that co-inoculation increased soybean grain yield by an average of 8.4%, accompanied by a 16.1% increase in nodule biomass and improved nitrogen and phosphorus uptake. Similarly, Steiner et al. [113] observed that co-inoculation of *Rhizobium tropici* and *A. brasilense* in common bean significantly increased nodulation, plant biomass, nitrogen accumulation, and grain yield compared with single inoculation. More recently, a meta-analysis conducted by Kaschuk et al. [114] confirmed that co-inoculation consistently enhances nodulation, root development, nitrogen accumulation, and grain yield across grain legumes, reinforcing the complementary roles of rhizobia and plant growth-promoting bacteria in sustainable crop production.

In Brazil, co-inoculation with *Rhizobium tropici* and *Azospirillum brasilense* in common beans and *Bradyrhizobium japonicum* and *A. brasilense* in soybeans was introduced in 2014 [4, 6]. The efficiency of this technology is based on the synergistic effect of the combination of beneficial microorganisms, which has the potential to promote significant increases in grain yield [4, 6]. The main mechanisms of action of this technology include N fixation (*Bradyrhizobium* spp. and *Rhizobium* spp.) and the stimulation and production of phytohormones (*Azospirillum* spp., *Pseudomonas* spp. and *Bacillus* spp.). The technology enhances the mechanisms of action of these microorganisms under the plants, resulting in higher yield increases than those observed with simple inoculation [115].

*Azospirillum* spp. is a successful case of co-inoculation, which has allowed other BPCPs to be developed and proven effective in co-inoculation trials. Examples include rhizobia with phosphate solubilizing bacteria (*Bacillus* spp.) [116], rhizobia with pathogen biocontrol bacteria (*Pseudomonas* spp. and *Bacillus* spp.), and rhizobia with bacteria that stimulate phytohormone production. In addition to the combination of rhizobia with bacteria that help with tolerance to abiotic conditions and/or the combination of microorganisms that have the same mechanisms of action, the following combinations are possible [117, 118].

The successful use of *Azospirillum* spp. in co-inoculation systems has stimulated the development of new microbial consortia combining rhizobia with other plant growth-promoting bacteria (PGPB) exhibiting complementary mechanisms of action [81, 83]. For example, co-inoculation of rhizobia with phosphate-solubilizing bacteria, particularly *Bacillus* spp., has been shown to improve phosphorus availability through the production of organic acids and phosphatases, enhancing phosphorus uptake, nodulation, biological nitrogen fixation, and grain yield [119, 120]. Likewise, microbial consortia including *Pseudomonas* spp. and *Bacillus* spp. contribute to biological control through the production of antibiotics, siderophores, lytic enzymes, and the induction of systemic resistance, thereby reducing pathogen incidence while promoting plant growth [83, 121]. In addition, co-inoculation with phytohormone-producing bacteria, particularly *Azospirillum brasilense*, stimulates root development through the synthesis of indole-3-acetic acid (IAA), gibberellins, and cytokinins, increasing root surface area, nutrient uptake, nodulation, and the efficiency of biological nitrogen fixation [81, 112]. These findings demonstrate that the success of co-inoculation depends on the complementary functional traits of the microbial partners, providing a strong scientific basis for the development of multifunctional microbial consortia for sustainable agriculture.

It should be noted that this combination of microorganisms takes into account the specificity of the plant and the microorganism [117, 118], which can lead to different effects. These microorganisms are used to meet nutritional requirements, protect against phytopathogens and tolerate abiotic stresses in plants. Some studies describe the benefits of combining bacteria with different mechanisms of action, as shown in Table 2.

**Table 2:** Benefits of combining strains of different microorganisms and their mechanisms of action in common bean

Microrganisms		Strains	Benefits
Biofertilizer	Biostimulator		
Rhizobium tropici (Rt)	Azospirillum brasilense (Ab)	Rt: SEMIA 4077 (CIAT-899) and Ab: Ab-V5	Co-inoculation provided an increase in the number of nodules and grain yield, in addition to presenting the greatest grain yield stability, producing around 3,439 kg ha-1 [4].
Rhizobium tropici (Rt)	Azospirillum brasilense (Ab)		Coinoculation provided a significant increase in nodulation and plant development, in addition to presenting greater grain yield stability, with an average production of 3,499 kg ha-1, which represents an increase of 15.4%, 14.8%, 11.0 % and 1.0% compared to the treatments inoculation with R. tropici, absolute control, registered product and nitrogen fertilization, respectively [2].
Rhizobium tropici (Rt)	Azospirillum brasilense (Ab)		Co-inoculation provided the highest average values ​​for gross revenue (2,471 US$ ha-1), net revenue (2,220 US$ ha-1) and benefit-cost ratio (1.4 US$ US$-1). In addition to presenting the lowest production cost (451 US$ ha-1), while the nitrogen fertilizer treatment with 80 kg ha-1 of N resulted in the highest average production cost (499 US$ ha-1) [52].
Rhizobium tropici (Rt)	Azospirillum brasilense (Ab)	Rt: SEMIA 4077 (CIAT-899) and Ab: Ab-V5 e Ab-V6	Co-inoculation resulted in return rates of 90% in Goiás and 114% in Minas Gerais, for commercial cultivation, and 13% in Goiás for family farming. For commercial and family farming, the production cost using N fertilizer is 5.0 and 8.5% higher, respectively [5].
			Co-inoculation showed an increase of about 9%, 25%, 35% and 31% in nodulation and plant development parameters, respectively, compared to the Rt treatment. These increases in nodulation and plant growth resulted in a yield of grains of 3,200 kg ha-1, which represented an increase of approximately 5% and 26% compared to nitrogen treatments and inoculation with R. tropici, respectively [7].
			The co-iconulation resulted in a grain yield of 2,892.42 kg ha-1, surpassing the nitrogen treatment, which was 2,741.91 kg ha-1. This result indicates that coinoculation can be an alternative to replacing nitrogen fertilizers, guaranteeing high levels of grain yield in beans [122].
Rhizobium tropici (Rt), Rhizobium leguminosarum (Rl) and R. freirei (Rf)	Azospirillum brasilense (Ab)	Rt: SEMIA 4077 (CIAT-899), Rl: SEMIA 4088, Rf: SEMIA 4080 and Ab: Ab-V5 e Ab-V6	Co-inoculation alleviates the negative effects of water stress and maintains the growth and grain productivity of common bean plants and, therefore, this management practice can be used by common bean producers to increase the drought tolerance of plants [113].
Rhizobium tropici (Rt), Rhizobium leguminosarum (Rl) and R. freirei (Rf)			Coinoculation positively influenced nodulation, plant development and grain yield of the bean cultivars BRS FC104 and BRS Notável [123].
Rhizobium tropici (Rt) and Bradyrhizobium diazoefficiens (Bd)	-	Rt: CIAT 899 (= SEMIA 4077) and Bd: CPAC 7 (= SEMIA 5080)	Co-inoculation can provide an increase in biological N fixation of between 50 and 60%, in addition to increasing the growth and grain yield of common beans [124].
Rhizobium leguminosarum (Rl)	Pseudomonas brassicacearum (Pb)	Rl: LCS0306 and Pb: RVPB2-2	The combination of microorganisms increased N2 fixation by 87% and productivity by 59%, compared to the uninoculated and unfertilized control. In addition to improving plant biomass and N-fixed, due to the indole-3-acetic acid (IAA production) and 1-aminocyclopropane-1-carboxylate (ACC) deaminase activity of P [8].
Rhizobium tropici (Rt), Bacillus subtilis (Bs) and Pseudomonas fluorescens (Pf)	Azospirillum brasilense (Ab)	Rt: CIAT 899 (= SEMIA 4077), Bs: CCTB04, Pf: CCTB03 and Ab: Ab-V5 e Ab-V6	Co-inoculations increased grain yield components associated with a 50% NPK dose, reducing the costs of synthetic fertilizers and reducing environmental impacts in the common bean production system [125].
Rhizobium tropici (Rt), Rhizobium spp. (R), Paenibacillus polymyxa (Pp) and Bacillus megaterium (Bm)	-	Rt: CIAT 899 (= SEMIA 4077), R1: IITA-PAU 987, R2: IITA-PAU 983, Pp: US and Bm: US	Co-inoculation increased nodule weight, plant development and increased N demand by 24%. Co-inoculation has a synergistic effect on bean development. The combined use of microorganisms improves the effectiveness of Rhizobium biofertilizers for common bean production [116].

*EN* Unidentified strain

In general, examples of combinations such as *Bradyrhizobium* sp, *Rhizobium* sp, *Azospirillum* sp, *Bacillus* spp. and *Pseudomonas* spp. can promote beneficial effects on plant development and increase the root system, resulting in higher grain yields [56]. When these microorganisms cannot be formulated into a single product, inoculants are marketed separately in kits. In the case of mixed inoculants, especially with rhizobia, it is necessary to ensure minimum concentrations according to legal standards and strict quality control. Depending on the microorganism, inoculants can be applied in the furrow or as foliar spray. Factors such as the lifestyle of the microorganisms, the method of application and soil management influence the efficiency of this technique. In Brazil, this practice has significantly increased the productivity of soybeans and common beans [4, 6, 114].

The success of co-inoculation is related to the synergistic effect of the compatibility of the mechanisms of action of the microorganisms. Several studies have highlighted the importance of co-inoculation from an agronomic point of view in common beans. In the work carried out by Souza and Ferreira [7] with *R. tropici* and *A. brasilense*, a positive effect was obtained, increasing the number of nodules, dry mass of nodules and dry mass of aerial parts by 9%, 25%, 35% and 31%, respectively, compared to the treatment with nitrogen fertilization, resulting in a grain yield of 3200 kg ha^− 1^, 5% and 26% higher than nitrogen fertilization and simple inoculation with *R. tropici*, respectively (Table 2). The same positive effect was found by Messias et al. [2], co-inoculation with *R. tropici* and *A. brasilense* provided an increase in nodulation and grain yield, as well as showing the greatest stability in grain yield, producing an average of 4,439 kg ha^− 1^ from the five sites evaluated, which represents an increase of 100, 93, 74 and 83 kg ha^− 1^ compared to nitrogen fertilization, registered product, simple inoculation with *R. tropici* and absolute control, respectively (Tabela 2).

From an economic point of view, Ferreira et al. [5] observed that co-inoculation resulted in returns of 90% and 114% in Goiás and Minas Gerais, respectively, and 13% in a family farming environment in the state of Goiás (Table 2). The same was observed by Messias et al. [52], with the best economic performance obtained by co-inoculating *R. tropici* and *A. brasilense*, which provided the highest average gross revenue (2,471 US$ ha^− 1^), net revenue (2,220 US$ ha^− 1^) and benefit-cost ratio (1.4 US$^−1^). In this study, co-inoculation resulted in the lowest production cost (451 US$ ha^− 1^), while nitrogen fertilization treatment with 80 kg ha^− 1^ N resulted in the highest average production cost (499 US$ ha^− 1^). In the study by Oliveira et al. [126], studying the coinoculation with *Rhizobium*, *Azospirillum* and microalgae in common bean, the coinoculalation of *Chlorella vulgaris* with *R. tropici* and *A. brasilense* resulted in grain yields of 1277.5 kg ha⁻¹ in clay soil and 2960.3 kg ha⁻¹ in sandy soil, representing an increase of 219.7 and 656.0 kg ha⁻¹, respectively, in relation to the double inoculation of *R. tropici* + *A. brasilense*. In sandy soil, common beans with triple coinoculation showed the highest profit (US$ 956 ha⁻¹), 25.6% higher than plants fertilized only with nitrogen.

Although several factors are considered critical for the efficiency of biological N fixation in bean, the use of commercial strains combined with genotypes with productive potential and good host specificity has resulted in high grain yields in excess of 4,000 kg ha^− 1^ [4]. However, some strategies have been adopted to ensure high yields without the use of nitrogen fertilizers, such as application methods and high doses of inoculants. In addition to maintaining the longevity of active nodules by correcting and improving soil fertility, including top dressing with copper (Cu), molybdenum (Mo) and nickel (Ni).

These benefits go beyond the economic aspect and have a positive impact on the environment by reducing or replacing the use of nitrogen, potassium and phosphate fertilizers, as well as reducing the use of insecticides and fungicides.

It is worth noting that the increased use of inoculants has also raised some scientific concerns, such as the identification of bacteria that, while promoting plant growth, may be harmful to human health [56]. Examples include *Enterobacter* spp. *Burkholderia cepacia* and *Pseudomonas aeruginosa* [1, 4, 56, 64, 105, 127], which cannot be recommended for inoculant production [105]. This highlights the importance of correct taxonomic identification of these microorganisms [105].

In the context of agronomic practices, the compatibility between fungicides used in seed treatment and the use of pre-inoculated seeds represents a significant limitation for the survival of bacteria [56, 128]. It is necessary to develop products that are compatible with cell protectors [56, 129] or to apply inoculants directly in the planting furrow, avoiding direct contact with the fungi [56, 109]. In addition, some farmers produce their own inoculants and biological control products under non-specialized conditions [56, 129], which can lead to the proliferation of human pathogens [56, 130, 131]. In this scenario, the quality of the product and the benefits of this technology are compromised [56].

The compatibility between seed-applied fungicides and microbial inoculants remains one of the major constraints to the successful establishment of beneficial microorganisms, as several active ingredients can markedly reduce bacterial survival on seeds and impair nodulation and biological nitrogen fixation [56, 128, 129, 132]. To overcome these limitations, considerable efforts have been directed toward developing inoculant formulations containing cell protectants and osmoprotectants capable of preserving bacterial viability during storage and after seed treatment. Compounds such as polyvinylpyrrolidone (PVP), glycerol, trehalose, sucrose, skim milk powder, sodium alginate, xanthan gum, and carboxymethyl cellulose (CMC) have been widely investigated because they reduce desiccation stress, stabilize cell membranes and proteins, improve bacterial adhesion to seeds, and minimize the toxic effects of pesticides [12, 103, 104]. Among these additives, PVP acts as a protective polymer by retaining moisture around bacterial cells and reducing oxidative stress, whereas glycerol functions as a compatible solute that stabilizes membrane integrity during dehydration. Likewise, alginate- and CMC-based formulations provide a physical matrix that prolongs cell survival and facilitates gradual bacterial release into the rhizosphere after sowing. In addition to formulation improvements, agronomic practices such as in-furrow inoculation or the temporal separation between seed treatment and inoculation have been shown to substantially increase bacterial survival by minimizing direct exposure to fungicides and insecticides [128, 132]. Collectively, these strategies improve inoculant performance under commercial production conditions and contribute to greater consistency in biological nitrogen fixation and crop productivity.

## Conclusions and perspectives

Inoculants containing rhizobia have been marketed for beans in Brazil for many years. More recently, inoculants containing other growth-promoting microorganisms have shown benefits in pest and disease control and water stress mitigation, and are increasingly gaining the confidence of growers.

These inoculants are currently being tested on bean crops with the aim of increasing the sustainability of the production system, promoting productivity gains, reducing environmental impacts and providing economic benefits. Despite the favorable scenario for the development of new inoculants, especially those containing consortia of microorganisms, it is crucial to improve the quality and formulation of these products. In addition, it is essential to ensure the compatibility of microorganisms with existing formulations to facilitate their use by growers.

Moreover, new combinations of microorganisms or consortia of microorganisms in common beans have attracted attention for their potential to enhance biological nitrogen fixation (BNF) and improve grain productivity. Some examples of these combinations include *Rhizobium* sp. with *Pseudomonas* sp., *Bacillus* sp., and the classic combination of *Rhizobium* with *Azospirillum* sp. The goal of these interactions is to explore the different mechanisms of action of microorganisms, functioning as biofertilizers, biostimulants, or biocontrol agents.

In this regard, strategies for using inoculants in common beans are focused on methods of inoculation and the dosages applied, whether through simple inoculation with *R. tropici* or through multifunctional inoculants with different mechanisms of action. These approaches have the potential to increase the adoption of inoculants in bean cultivation. However, further in-depth studies are needed to understand the interaction mechanisms and the benefits resulting from the combined application of microorganisms. Establishing response patterns to biotic and abiotic factors will be essential to ensure the efficiency of these technologies in the field, especially considering the edaphoclimatic variations of each region. This will not only allow for increased productivity but also reduce the use of nitrogen, phosphorus, and potassium fertilizers, as well as decrease the need for fungicides.

Therefore, future research should focus on technological advancement, promoting the adoption of new sustainable practices that result in productivity gains and a positive economic and environmental impact on common bean production systems in Brazil and other tropical countries. 

## Data Availability

The datasets generated during and/or analyzed during the current study are available from the corresponding author on reasonable reques.
